# Renaming *Candida glabrata*—A case of taxonomic purity over clinical and public health pragmatism

**DOI:** 10.1371/journal.ppat.1012055

**Published:** 2024-03-15

**Authors:** David W. Denning

**Affiliations:** Manchester Fungal Infection Group, The University of Manchester and Manchester Academic Health Science Centre, Manchester, United Kingdom; University of Maryland, Baltimore, UNITED STATES

In recent years, many medical mycologists have adopted the proposed name change of *Candida glabrata* to *Nakaseomyces glabrata* and some to *Nakaseomyces glabratus*. Is this helpful or a hindrance? I argue it is unhelpful.

## Is *Candida glabrata* an important pathogen?

*Candida glabrata* is the third or fourth most common human pathogen among *Candida* spp. [1]. It is a haploid yeast. It causes candidaemia, invasive candidiasis and candiduria in adults, and rarely in children. The most recent estimate of the annual incidence of all candidaemia and invasive candidiasis is approximately 1.5 million [2], of which *C*. *glabrata* is probably responsible for about 25%. It is the second most common cause of vulvovaginal candidiasis and is especially prominent in recurrent cases (RVVC). Globally an estimated 135 million women are affected by RVVC [3], so about 13 million are likely infected with *C*. *glabrata*. Its colonial morphology is small and unlike most other pathogenic, *Candida* spp. it does not usually produce pseudohyphae or true hyphae. It grows well in anaerobic blood culture media, but more slowly than *C*. *albicans* or *C*. *tropicalis* and forms biofilms. It is often resistant to fluconazole, depending on where the breakpoint is set. A simple search of PubMed with “*Candida glabrata*” yields 7,000 references.

## How did *Candida glabrata* get its name?

In the early days of molecular biology and fungal taxonomy in 1977 Frank Odds, Michael Rinaldi, Chester Cooper, Annette Fothergill, Lester Pasarell, and Michael McGinnis recognised that *Torulopsis glabrata* was better retained in the *Candida* genus, even though its taxonomy placed it among the *Saccharomyces* species [4]. As a “new kid on the mycology block” I remember asking Frank about this and he argued that it was better for clinical practice that glabrata was grouped within the *Candida* spp.

The following year, David Yarrow and Sally Meyer proposed abandoning the genus *Torulopsis* and renaming multiple species as *Candida* species, including *C*. *glabrata* [5]. Their entry reads: “*Candida glabrata* (Anderson) Meyer et Yarrow comb. nov. Basionym: *Cryptococcus glabratus* Anderson-J. Infect. Dis. 21:379,1917.”

The genus *Nakaseomyces* was introduced in 2003 to honor the contributions to taxonomy of Dr. Takashi Nakase by Cletus Kurtzmann, including recognition that *C*. *glabrata* was appropriately categorised in this genus, but was not renamed [6].

When whole genome sequencing became available (2009), Geraldine Butler, Christine Cuomo, and many others grouped *C*. *glabrata* in with 7 other *Candida* genomes with the clear recognition that it fitted squarely in the middle of the *Saccharomyces* genus (as it was called then) from a taxonomic perspective [7]. Mary Brandt and Shawn Lockhart maintained *C*. *glabrata* in their 2012 review [8]. Tony Gabaldon and many colleagues continued to refer to the *Candida glabrata* clade in their comparative genomics paper of *Candida* spp. in 2013 [9].

## When did the proposed *Nakaseomyces glabrata* name emerge?

Andrew Borman and Elizabeth Johnson published a review of name changes of Fungi of Medical Importance in 2021 and argued the case for renaming *C*. *glabrata* as *Nakaseomyces glabrata* [10]. This was followed by a formal proposal for renaming to *Nakaseomyces glabratus* from Masako Takashima and Takashi Sugita in 2022 [11], reverting to the original species name used by Harry Anderson, when he described the species grown from human faeces in 1917 [12].

## Aside from taxonomic nomenclature rules, what are the arguments for renaming *Candida glabrata*?

Borman and Johnson opined “Thus, we believe that the practice of employing revised names for these pathogenic yeast species will be more informative to the clinician than persisting with the current misleading practice of using historical genera to group hundreds of genetically distantly related yeast species.” For *C*. *glabrata*, I disagree.

## What are the downstream consequences of a name change to *Nakaseomyces glabrata*?

Those advocating for this name change after 25 years of nomenclature stability have not properly considered the disruption to clinical disease terminology in enough detail and the negative impact on public health for medical mycology. Long established terms such as invasive candidiasis and candidaemia become unworkable, as *C*. *glabrata* is such a common cause of these syndromes. As *C*. *glabrata* is a cause of about 10% of RVVC, this term also becomes unworkable. Non-culture-based diagnostics for life-threatening *Candida* infection, such as beta-1.3-D glucan, detect *C*. *glabrata* as well as other *Candida* spp. and clinical interpretation would be compromised by the proposed nomenclature change. Given that medical textbooks are revised infrequently with long gaps, any taxonomic changes lead to years of uncertainty and potential confusion for clinicians and students.

**Fig 1:**
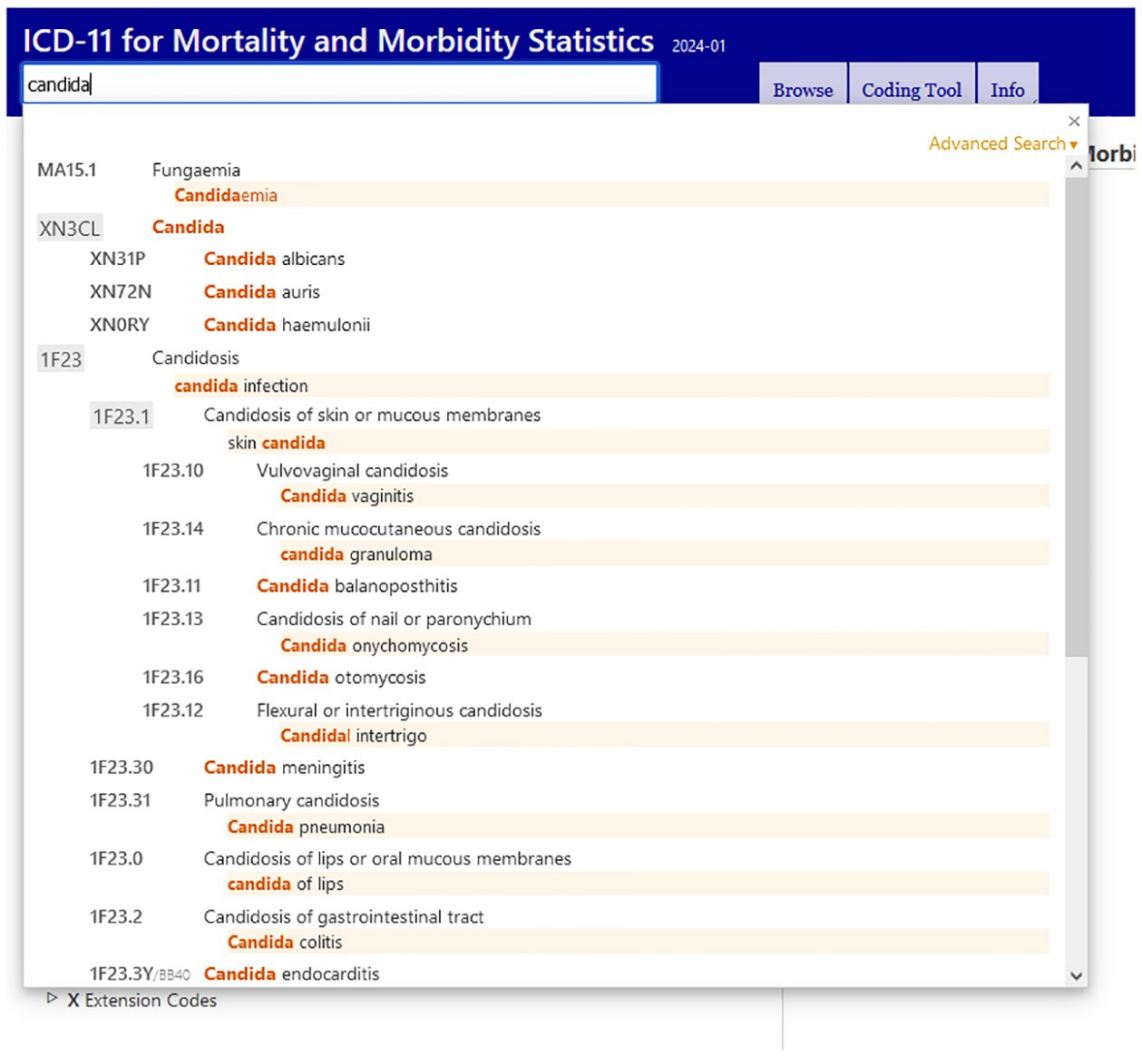
International Classification of Disease 11, version January 2024.

The International Classification of Disease (ICD) of *Candida* infections would need revising again if *N*. *galbratus* or *N*. *glabrata* remains in common parlance (Fig 1) [13]. ICD11 has no entry for *Nakaseomyces* spp.

Antifungal drug registrations for the azoles, echinocandins, ibrexafungerp, and otesaconazole are for *Candida* infections. For example, the US Federal Drug Administration (FDA) licence of caspofungin for clinical use is: “Treatment of Candidemia and the following *Candida* infections: intra-abdominal abscesses, peritonitis, and pleural space infections” and “Treatment of Esophageal Candidiasis” [14]. Ibreaxafungerp is licenced by the FDA for “the treatment of adult and post-menarchal pediatric females with vulvovaginal candidiasis (VVC)” [15]. Renaming of such an important human pathogen possibly renders the indications for these drugs invalid at worst and requiring revision at best, in every country where antifungal agents active against *Candida* spp. are approved. Approvals and usage of over the counter agents for the treatment of VVC may also be rendered invalid or require revision, depending on the patient information provided.

From a public health perspective, the collection of incidence and susceptibility data of *Candida* infections remains of paramount importance for multiple reasons. The splitting of *C*. *glabrata* away from the rest of the main *Candida* pathogens is unhelpful from a numerical perspective. It dilutes the importance of *Candida* as a major human group of pathogens probably responsible for close to a million deaths annually [2]. For women affected by yeast infections, especially RVVC, understanding of their condition and its management becomes unnecessarily complicated.

As a group fungal disease is under-recognised, under-diagnosed, under-treated, and under-funded. Splitting off additional major pathogenic species from well-established disease concepts engenders uncertainty, difficulties in messaging and hampers advocacy. Those in diagnostic laboratories who advise clinicians and antifungal stewardship teams will have an even harder time explaining the subtleties of results and treatment options to many clinicians who have barely heard of *Candida*.

The international code of nomenclature for algae, fungi, and plants leaves open the option to retain both species and genus names for well-argued reasons. De Hoog and many knowledgeable co-authors left open the possibility to retain the name *Candida glabrata* in their sensible advice on nomenclatural stability recommendations for fungi [16]. So in my view, Takashima and Sugita’s proposed nomenclature change from *C*. *glabrata* to *N*. *glabratus* should be rejected. I agree with Frank Odds’ (and many others’) view that it is better to persist with *C*. *glabrata* for clinical use and in medical mycology.
